# Serum Vitamin D in Children with Hemophilia A and Its Association with Joint Health and Quality of Life

**DOI:** 10.3390/hematolrep16040071

**Published:** 2024-11-26

**Authors:** Aida M. S. Salem, Takwa Mohamed AbdEltwwab, Hanan Hosni Moawad, Marwa O. Elgendy, Reham S. Al-Fakharany, Ahmed Khames, Mohamed Hussein Meabed

**Affiliations:** 1 Department of Pediatrics, Faculty of Medicine, Beni-Suef University, Beni-Suef 62521, Egypt; aida.mohamed@med.bsu.edu.eg (A.M.S.S.); m1hmeabed2@med.bsu.edu.eg (M.H.M.); 2 Department of Pediatrics, General Authority for Health Insurance, Beni-Suef 62511, Egypt; 3 Department of Clinical and Chemical Pathology, Faculty of Medicine, Beni-Suef University, Beni-Suef 62521, Egypt; hananhosni52@yahoo.com; 4 Department of Clinical Pharmacy, Beni-Suef University Hospitals, Faculty of Medicine, Beni-Suef University, Beni-Suef 62521, Egypt; 5 Department of Clinical Pharmacy, Faculty of Pharmacy, Nahda University (NUB), Beni-Suef 62764, Egypt; 6 Department of Medical Physiology, Faculty of Medicine, Beni-Suef University, Beni-Suef 62521, Egypt; drrehamelfakharany@gmail.com; 7 Department of Pharmaceutics and Industrial Pharmacy, College of Pharmacy, Taif University, Taif 21944, Saudi Arabia; a.khamies@tu.edu.sa

**Keywords:** hemophilia A, vitamin D, HJHS, Haemo-Qol

## Abstract

**Background/Objectives**: Hemophilia A is an X-linked recessive illness produced by a deficiency of coagulation factor VIII. This study aimed to evaluate serum vitamin D in hemophilic pediatric patients and its correlation with joint health and quality of life. **Methods**: This case-control study was performed on ninety children under the age of 18 years old and separated into two groups: study group of 45 children with hemophilia A and control group of 45 healthy children at an outpatient pediatric hematology clinic at the Beni-Suef University hospitals. **Results**: Serum vitamin D levels were significantly lower in hemophilia A patients than in controls (*p* < 0.001). The level of serum vitamin D was deficient in 38 (84.4%), insufficient in 4 (8.8%) and sufficient in 3 (6.6%) in the study group while deficient in 8 (17.7%), insufficient in 16 (35.5%) and sufficient in 21 (46.6%) in the control group. Total hemophilia joint health score (HJHS) had a significant negative correlation with serum total calcium (R = −0.31, *p* = 0.038) and serum vitamin D level (R = −0.974, *p* < 0.001) while also positively correlated with alkaline phosphatase (R = 0.834, *p* < 0.001). A quality-of-life index that is specific to total hemophilia (Haemo-Qol/Haem-A-QoL) had a significant positive correlation with total hemophilia joint health score (HJHS) (R = 0.934, *p* < 0.001) and negatively correlated with serum vitamin D level (R = −0.924, *p*-value lower than 0.001), alkaline phosphatase (R = 0.842, *p* < 0.001), and severity of hemophilia (R = 0.67, *p* < 0.001). **Conclusions**: patients with hemophilia A had lower vitamin D levels than healthy controls. The severity of vitamin D deficiency is related positively to (HJHS) hemophilia and quality of life hemophilia cases according to Haemo-QoL.

## 1. Introduction

Hemophilia is an X-linked inherited disorder characterized by a chronic, life-threatening condition caused by low levels of factor IX (FIX) in hemophilia B and coagulation factor VIII (FVIII) in hemophilia A [1]. Hemophilia A accounts for about 80% of cases, while hemophilia B makes up the remaining 20%. Patients are classified based on their residual levels of FVIII/FIX: those with levels <1 IU/dL have severe hemophilia, accounting for nearly half of all cases; those with 1–5 IU/dL have moderate hemophilia; and those with >5 IU/dL have mild hemophilia. Even among severe cases, the bleeding phenotype can vary significantly [2].

Key symptoms of hemophilia include excessive bleeding during surgery (such as circumcision), spontaneous intracranial bleeding in newborns, atraumatic painful hemarthrosis, unexplained bruising when crawling or walking begins, and excessive musculocutaneous hemorrhage that can occur spontaneously or after intramuscular vaccination [3].

Hemarthrosis, also known as hemophilic arthropathy, is the primary morbidity associated with joint hemorrhage and one of the most negative effects of hemorrhagic events.

Nearly half of hemophilia patients suffer from hemophilic arthropathy [4], which results from recurrent hemarthrosis and leads to progressive and degenerative joint damage, causing moderate to severe disability and significantly impacting the patient’s quality of life [5].

Frequent immobilization due to arthropathy-induced incapacity is a key risk factor for decreased bone density [6]. The fear of bleeding limits mobility and weight-bearing activities, further reducing bone mineral density (BMD) [6]. Long-term immobility, along with Hepatitis C and HIV, contributes to the reduction in BMD. Chronic inflammation from these infections adversely affects bone metabolism and modulates the bone-remodeling pathway through pro-inflammatory cytokines like TNF-a, IL-1, IL-6, and IL-17, promoting osteoclast differentiation and leading to an imbalance between bone resorption and formation, resulting in osteoporosis and fractures [7]. Subclinical 25(OH)D deficiency is also associated with osteoporosis and low BMD [8]. Living with hemophilia significantly impacts health-related quality of life due to the economic burden of medical care, frequent bleeding episodes, and psychological health challenges [8].

Few studies have explored the role of vitamin D in children with hemophilia and with inconsistent findings. Some researchers have observed lower 25(OH)D levels in hemophilia patients compared to healthy controls, while others have found no difference [9]. This study aimed to evaluate serum vitamin D levels in pediatric hemophilia patients and their correlation with joint health and quality of life.

## 2. Materials and Methods

This case-control study was conducted on ninety children under 18 years old, divided into two groups: a study group of 45 children with hemophilia A and a control group of 45 healthy children. The study took place at the outpatient pediatric hematology clinic in Beni-Suef University Hospitals from July 2021 to September 2022.

The research included children diagnosed with coagulation factor VIII deficiency (hemophilia A), categorized based on factor VIII activity as follows: mild (>5 units/dL), moderate (1–5 units/dL), or severe (<1 unit/dL), as defined by Arnold and Hilgartner [10]. This classification included both male and female participants under 18 years of age.

Children with thyroid or parathyroid diseases, a history of chronic renal diseases, gastrointestinal diseases, hepatic diseases, or those using steroidal anti-inflammatory medicines, antiepileptic drugs, or any other medication affecting bone metabolism were excluded from the study.

All participants underwent a comprehensive evaluation, including a full medical history, anthropometric measurements, and complete clinical examinations.

### 2.1. Haemophilia Joint Health Score (HJHS)

Version 2.1 of the HJHS, a new joint scoring tool, was used to assess joint impairment. This tool evaluates early signs of arthropathy in the six major joints: knees, elbows, and ankles. It includes the following components: crepitus on motion, muscle atrophy, duration of swelling, loss of range of motion in flexion and extension, strength, global gait, and joint pain. The HJHS total score is obtained by combining the joint total scores and the global gait score, with a score of zero indicating optimal joint health and 124 indicating poor joint health.

### 2.2. Hemophilia-Specific Quality of Life Index (HaemoQoL and Haem-A-QoL Questionnaires) [11]

The Haem-A-QoL instrument is the disease specific questionnaire to assess QoL of adult patients with haemophilia. This instrument has 10 dimensions and 46 items (dimensions: physical health, feelings, view, sport and leisure time, work and school, dealing, treatment, future, family planning, and relationships/partners.

Score of each dimension as well as the total score is transformed to a scale from 0 to 100. Higher scores represent the worst HRQoL. 

To assess QoL of children and adolescents with hemophilia, the long version of Haemo-QoL questionnaire was used after approval and permission of the Haemo-QoL Group. The younger children’s version (age group I: 4–7 years) of the questionnaire has 21 items comprising 8 dimensions (physical health, feelings, view, family, friends, others, sport and school/kindergarten, treatment). The school children’s version (age group II: 8–12 years) contains 2 additional domains (received support and dealing) with overall 64 items, and the adolescents’ version (age group III: 13–16/18 years) has 2 further additional domains (relationships, future) with 77 items. In addition to the Haem-AQoL, the score of each dimension and the total score was recorded. 

Haemo-QoL are transformed to a 0 to 100 scale, and the higher scores indicate the worst HRQoL.

### 2.3. Determination of Serum Vitamin D

In order to determine the vitamin D status, serum 25(OH)D3 levels were measured using an enzyme-linked immunoassay (ELISA).

### 2.4. Assay Procedure

Twenty microwells should be filled with calibrators, controls, and test samples, each containing 20 µL. In each well, introduce 100 µL of vitamin D assay buffer. Protect the plate from light with aluminum foil or another material, then incubate it at room temperature in a static environment for thirty minutes. With caution, remove the foil covering each well and proceed to add twenty-five microliters of biotinylated vitamin D analogue. Securely seal the wells in the plate, shield from light with foil or another suitable material, and incubate at room temperature in an inactive environment for one hour. Five times through, dispense 350 L of working washing solution into each well, ensuring that the contents are fully aspirated. Incorporate 100 µL of Streptavidin-HRP into every well. Cover the plate wells with foil or another light-resistant material and securely seal. Allow the plates to incubate at an ambient temperature for a duration of thirty minutes. Five times through, dispense 350 L of working washing solution into each well, ensuring that the contents are fully aspirated. Incorporate one hundred microliters of TMB reagent into each well. With the plate covered with aluminum foil, incubate static at room temperature for twenty minutes. Add one hundred microliters of ELISA Stop Solution to each well without delay. Mix with care. Obtain the absorbance at 450 nm after a duration of ten minutes.

### 2.5. Ethical Considerations

Parental consent was obtained for the study. The parents of the cases who participated in the investigation provided written informed consent subsequent to receiving authorization from the Local Ethical Committee. 

Approval No: FBBSUREC/06062021/Abdeltawwab.

### 2.6. Statistical Methods

The statistical program for social science (SPSS) will be utilized to analyze the data. In terms of standard deviation, mean, median, and range, the quantitative variables will be identified. Frequency and percentage values will be utilized to describe qualitative variables when applicable. Qualitative variables that adhere to a normal distribution will be correlated via person correlation. Serum vitamin D concentration in children with hemophilia A and its association with joint health and quality of life will be determined using a receiver operating characteristic (ROC) curve. When the calculated *p* value is fewer than 0.05, it is non-significant; otherwise, it is significant, and if it is fewer than 0.05, it is highly significant.

## 3. Results

In the study group, the mean age was 8.70 ± 3.62 years, while in the control group, it was 9.82 ± 3.08 years. Both groups were matched for age and sex (Table 1).

Among the 45 participants in the study group, 42 were from rural areas and 3 were from urban areas. Among the control group, 45 were from rural areas. A family history of similar conditions was present in 21 patients (46.7%), while 24 patients (53.3%) had no family history (Table 2).

The number of factor infusion per month was 3.33 ± 0.92 ranging from 1 to 4 times. The factor level in our study group was severe (<1 IU/dL) in 30 (66.7%), moderate (1–5 IU/dL) in 13 (28.9%), and mild (>5 IU/dL) in 2 (4.4%). Antibodies against factor 8 were detected in only 8 (17.8%) who received primary prophylaxis (Emicizumab) (Table 3).

The number of bleeding events per month ranged from 1 to 7 times per month in our study group while the number of joint bleedings per month ranged from 1 to 4 times (Table 4).

The investigation group had significantly lower serum vitamin D levels (ng/mL) compared to the control group (*p* < 0.001), while serum alkaline phosphatase levels were significantly higher in the study group than in the control group (*p* < 0.001). However, there was no statistically significant difference between the study and control groups in terms of serum total calcium (*p* = 0.108) and serum phosphorus (*p* = 0.195) (Table 5).

Vitamin D deficiency was much more prevalent in the investigation group compared to the control group (84.4% vs. 17.7%, respectively) (*p* < 0.001). In the study group, 8.8% had insufficient serum vitamin D levels, while 6.6% had sufficient levels. In contrast, in the control group, 35.5% had insufficient levels, and 46.6% had sufficient levels (Table 6).

**Figure 1:**
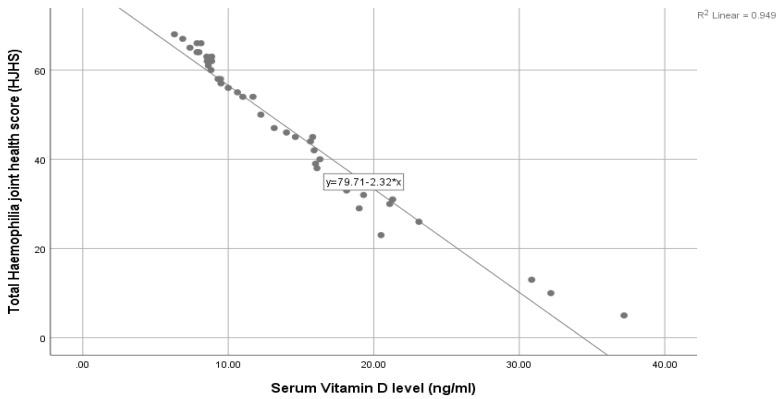
Association between total hemophilia joint health score (HJHS) and serum vitamin D level (ng/mL).

Total hemophilia joint health score (HJHS) showed a significant negative correlation with serum total calcium (R = −0.31, *p* = 0.038) and serum vitamin D level (R = −0.974, *p* < 0.001), while it was positively correlated with alkaline phosphatase (R = 0.834, *p* < 0.001). (Table 7, Table 8 and Figure 1).

A quality-of-life index that is specific to total hemophilia (Haemo-Qol/Haem-A-QoL) had significant positive correlation with total hemophilia joint health score (HJHS) (R = 0.934, *p*-value lower than < 0.001), alkaline phosphatase (R = 0.842, *p*-value lower than 0.001) and severity of hemophilia (R = 0.67, *p*-value lower than 0.001). Conversely, it was negatively correlated with serum vitamin D level (R = −0.924, *p* < 0.001) and showed no association with serum phosphorus (R = 0.261, *p* = 0.099) and serum total calcium (R = −0.115, *p* = 0.474) (Table 9).

## 4. Discussion

Vitamin D deficiency is particularly necessary for bone health in individuals with hemophilia, especially children with severe hemophilia [12]. Our study’s demographic data revealed that most patients came from rural areas, with fewer from urban areas. This disparity may be attributed to the lack of blood analysis, family screening for hemophilia during marriage, or postnatal examination for hemophilia detection in rural regions.

This observation aligns with research by Singh et al., which found that 159 (41.30%) cases were from urban areas, while 226 (58.70%) were from rural areas [13]. These results suggest that factor activity in hemophilia patients may vary across different racial populations due to the influence of geographical distribution.

Our findings indicated that the control group had significantly higher serum vitamin D levels (29.05 ± 8.76 ng/mL) compared to the study group (14.75 ± 6.90 ng/mL) (*p* < 0.001). In the hemophilic group, 84.4% versus 17.7% were deficient in vitamin D (25(OH)D < 20 ng/mL) (*p* < 0.001). This variance was not observed in the control group.

These results are consistent with the research by Abbasnezhad et al. which found that vitamin D deficiency affected 48.3% of adults and 77.8% of adolescents with hemophilia. Furthermore, their study showed that the average serum concentrations of 25(OH)D were significantly lower in children with hemophilia compared to healthy controls of the same age and sex (19.97 ± 2.61 ng/mL vs. 22.11 ± 4.21 ng/mL, respectively, *p* < 0.05). However, no significant difference in 25(OH)D levels was observed between hemophilic and healthy adults (22.06 ± 4.47 ng/mL vs. 22.38 ± 3.85 ng/mL, respectively) [7].

Our results showed that serum alkaline phosphatase levels were significantly higher in the investigation group compared to the control group (186.11 ± 93.71 ng/mL vs. 121.86 ± 60.54 ng/mL, respectively) (*p* < 0.001). There was no statistically significant difference between the control and study groups in terms of serum total calcium (*p* = 0.108) and serum phosphorus (*p* = 0.195).

These findings are consistent with the study by Alioglu B et al. which found no significant difference between children with severe hemophilia and control groups regarding serum calcium (8.9 ± 1.1 mg/dL vs. 9.1 ± 1.2 mg/dL, respectively) and serum phosphorus (4.2 ± 1.1 mg/dL vs. 4.3 ± 1.8 mg/dL, respectively) [14].

Our study also examined the association between the total hemophilia joint health score (HJHS) and levels of serum phosphorus, serum total calcium, alkaline phosphatase, and serum vitamin D. We found that the total HJHS had a significant negative correlation with serum total calcium (R = −0.31, *p* = 0.038) and serum vitamin D levels (R = −0.974, *p* < 0.001), while it was positively correlated with alkaline phosphatase (R = 0.834, *p* < 0.001).

Alioglu et al. also reported a significant inverse correlation between serum 25(OH)D levels and the total joint score in hemophilic patients. Another study found that phosphorus and serum 25(OH)D levels had a significant negative correlation with joint dysfunction and a significant positive correlation with functional activity, particularly in adolescents with hemophilia. [14] It is well established that serum trace minerals, as metalloenzymes, are crucial in the formation of proteins and collagen, which are essential for bone and joint health [7,15].

Additionally, our results showed that serum vitamin D levels were significantly positively correlated with the age of diagnosis (R = 0.567, *p* < 0.001) and the age of first joint bleeding (R = 0.382, *p* < 0.001).

Similarly, Sanadhya and Singh found a statistically significant association between serum vitamin D levels and the severity of hemophilia [16]. A previous study indicated that 97.14% of patients with severe hemophilia had inadequate vitamin D levels, suggesting a correlation between the severity of hemophilia and the severity of vitamin D deficiency.

We found that the quality-of-life index specific to hemophilia (Haemo-Qol/Haem-A-QoL) was negatively correlated with serum vitamin D levels (R = −0.924, *p* < 0.001), particularly in areas related to physical health, sports activity, and feelings of loneliness and embarrassment. Additionally, the hemophilia-specific quality of life index showed a significant positive correlation with the total hemophilia joint health score (HJHS) (R = 0.934, *p* < 0.001).

Vitamin D deficiency should be tested in all individuals with hemophilia for early diagnosis and treatment. Low-dose prophylaxis in severe hemophilia helps preserve bone mineral density and increases vitamin D levels [17].

Egyptian children with hemophilia require dedicated efforts from pediatricians and psychiatrists to enhance their quality of life [18].

To assess quality of life, we used the Haemo-QoL, a standardized and validated self-report questionnaire for children. It has two versions for different age groups: version I for children aged four to seven years and version II for children aged eight and older. Version I includes six domains: physical health, feelings, self-perception, school and sports, friends, and treatment. Version II expands to eight domains, adding dealing and perceived support. In the Haemo-QoL questionnaires, a higher total score indicates a poorer quality of life [19].

## 5. Conclusions

Hemophilia patients exhibit lower serum vitamin D levels and higher serum alkaline phosphatase levels compared to healthy controls, though their total calcium and phosphorus levels remain normal. Patients with greater joint involvement have higher total hemophilia joint health scores (HJHS), which are positively correlated with the severity of vitamin D deficiency. Additionally, children with hemophilia A have a poorer quality of life compared to their healthy peers, and the total hemophilia-specific quality of life index (Haemo-Qol/Haem-A-QoL) shows a negative correlation with serum vitamin D levels.

## Figures and Tables

**Table 1:** Age distribution and residence of both groups.

	Study Group (*n* = 45)	Control Group (*n* = 45)	Test of Sig.	*p*-Value
Age (years)				
(Min.–Max.)	(1–16)	(5–17)		
Mean ± SD	8.70 ± 3.62	9.82 ± 3.08	t = −1.59	0.113
Residence				
Rural	N:42 (93.3%)	N:45 (100%)	t = 3.1	0.242
Urban	N:3 (6.7%)	N:0 (0%)		

**Table 2:** Demographic data of the study group (*n* = 45).

Parameter	N	%	Test-Used	Significance
Residence				
Urban	3	6.7	χ^2^ = 5.57	0.0012 **
Rural	42	93.3		
Family history of similar condition				
No	24	53.3	χ^2^ = 3.2	0.02
Yes	21	46.7		
Consanguinity				
No	27	60	χ^2^ = 3.15	0.02
Yes	18	40		

** refers to high significance.

**Table 3:** Factor level of the study group.

Parameter	N	%	Test-Value	Significance
The severity of hemophilia (Factor level IU/dL)				
Mild > 5	2	4.4	χ^2^ = 4.28	0.022 *
Moderate 1–5	13	28.9		
Severe < 1	30	66.7		
Antibodies against factor 8				
Detected	8	17.8	χ^2^ = 4.46	0.024 *
Not detected	37	82.2		
Primary prophylaxis				
No	37	82.2	χ^2^ = 3.22	0.05 *
Emicizumab(Hemilibra)	8	17.8		

* refers to significance.

**Table 4:** Clinical data of the study group.

Variables	N = 45
Number of bleeding events per month	
Min.–Max.	1.00–7.00
Mean ± SD	3.689 ± 1.24
Number of joint bleedings per month	
Min.–Max.	1.00–4.00
Mean ± SD	2.5333 ± 0.94388
Frequency of joint bleeding per month	N (%)
1 time	5 (11.1%)
2 times	20 (44.5%)
3 times	11 (24.4%)
4 times	9 (20%)

**Table 5:** Comparison between the study group and control group as regards serum total calcium (mg/dL), alkaline phosphatase (U/L), serum phosphorus (mg/dL), and serum vitamin D level (ng/mL).

Group	Study Group				Control Group				*p*
	Range	Mean	±	SD	Range	Mean	±	SD	
Serum Total Calcium (mg/dL)	5.87–10.67	9.28	±	1.12	5.85–10.98	9.73	±	0.68	0.108
Serum Phosphorus (mg/dL)	3.41–8.40	5.69	±	1.49	3.45–8.47	5.27	±	1.08	0.195
Alkaline Phosphatase (U/L)	81–365	186.11	±	93.71	70–301	121.86	±	60.54	<0.001 **
Serum Vitamin D level (ng/mL)	6.30–37.20	14.57	±	6.90	6.35–38.26	29.05	±	8.76	<0.001 **

Mann–Whitney U Test, *p*: *p*-value for comparing between the studied groups, ** = Significant at (*p* < 0.001).

**Table 6:** Serum vitamin D status in the control group and study group.

	Study Group *n*, (%)	Mean ± SD	Control Group *n*, (%)	Mean ± SD	*p*-Value
Deficient < 20 ng/mL	38 (84.4%)	12.35 ± 4.11	8 (17.7%)	18.39 ± 0.97	0.0002 **
Insufficient ≥ 20 to > 30 ng/mL	4 (8.8%)	21.5 ± 1.11	16 (35.5%)	24.3 ± 3.34	0.1308
Sufficient ≥ 30 ng/mL	3 (6.6%)	33.4 ± 3.35	21 (46.6%)	36.71 ± 5.89	0.3575

** = Significant at (*p* < 0.001).

**Table 7:** Association between total hemophilia joint health score (HJHS) and serum total calcium, alkaline phosphatase, serum phosphorus, serum vitamin D level (ng/mL).

		Total Hemophilia Joint Health Score (HJHS)
Serum Total Calcium	r	−0.31
	*p*	0.038 *
Serum Phosphorus	r	0.261
	*p*	0.083
Alkaline Phosphatase	r	0.834
	*p*	<0.001 **
Serum Vitamin D level (ng/mL)	r	−0.974
	*p*	<0.001 **

* refers to significance; ** refers to high significance.

**Table 8:** Mean ± standard deviation and international consistency of Haemo-QOL and Haemo-A-QOL for different age groups.

Variables	Group I 4–7 Years (*n* = 14)	Group II 8–12 Years (*n* = 24)	Group III 13–16 Years (*n* = 3)	Test of Significance	*p* Value
	Mean ± SD	Mean ± SD	Mean ± SD		
Physical health	18.071 ± 5.877	16.042 ± 6.617	12.667 ± 8.021	F = 1.004	0.376
Feelings	5.786 ± 1.805	6.542 ± 3.050	4.000 ± 1.732	F = 1.398	0.260
View of yourself score	4.357 ± 1.781	6.167 ± 2.615	4.667 ±2.082	F = 2.832	0.071
Sports and school score	14.429 ± 4.702	10.625 ± 5.282	7.667 ± 3.512	F = 3.601	0.037 *
Family score	4.857 ± 1.748	5.917 ± 2.586	4.000 ± 2.000	F = 1.546	0.226
Friends score	2.000 ± 0.555	3.083 ± 1.816	2.667 ± 2.082	H = 5.524	0.063
Treatment score	2.071 ± 0.829	5.417 ± 2.701	4.333 ± 1.528	H = 13.507	0.001 *
Dealing score	--	3.708 ± 2.053	2.000 ± 1.000	H = 25.727	<0.001 *
Perceived support score	--	4.000 ± 2.284	2.667 ± 2.309	H = 22.648	<0.001 *
Total hemophilia specific Quality of life index (Haemo-Qol)	51.571 ± 15.441	61.500± 25.447	44.667 ± 24.111	F = 1.343	0.273

* refers to significance.

**Table 9:** Correlation between indicator of quality of life that is particular to hemophilia (Haemo-Qol/Haem-A-QoL) and total hemophilia joint health score (HJHS), serum vitamin D, and assessed minerals.

		Total Hemophilia Specific Quality of Life Index (Haemo-Qol/Haem-A-QoL)
Total hemophilia joint health score (HJHS)	r	0.934
	*p*	0.000 **
Serum total calcium	r	−0.115
	*p*	0.474
Serum phosphorus	r	0.261
	*p*	0.099
Alkaline phosphatase	r	0.842
	*p*	0.000 **
Serum vitamin D level (ng/mL)	r	−0.924
	*p*	0.000 **

** = Significant at (*p* < 0.001).

## Data Availability

The datasets used and/or analyzed during the current study are available from the corresponding author on reasonable request.
